# Pioneering new models to drive innovation in R&D and commercialization for breast cancer inventions

**DOI:** 10.1242/dmm.015156

**Published:** 2014-02


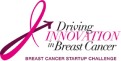


In September 2013, the Avon Foundation, the NIH-funded National Cancer Institute (NCI) and The Center for Advancing Innovation announced their partnership to create the first Breast Cancer Start-up Challenge (http://www.breastcancerstartupchallenge.com), a global competition that aims to advance biomedical inventions by launching start-ups. The challenge features a set of ten unlicensed breast cancer inventions (nine from the NCI intramural program and one from an Avon Foundation grantee) that have commercial viability and the potential to improve public health. The primary goal is to stimulate the creation of start-up businesses based on these inventions. Teams – made up of students led by established entrepreneurs – are competing to devise business plans; their efforts will be judged by an expert panel, including, among many other leaders in the pharmaceutical industry, George F. Tidmarsh, MD, PhD, CEO of La Jolla Pharmaceutical Co. and Consulting Editor of *Disease Models & Mechanisms* (DMM), Michael King Holly, Pharm. D, Senior Vice President of Quintiles Innovation, and Katherine Bowdish, PhD, Vice President R&D and Head of Sunrise, Sanofi.

Highlighting its status as one of the first initiatives of its kind, this contest truly has a number of ‘firsts’. Indeed, it is the first time that:

1. a therapeutically oriented foundation has sponsored a contest to launch start-ups
2. a Federal lab has put their inventions in an international, university-based contest
3. the inventions will be directly informed by the result of assessing over 4000 inventions in a Federal lab and across 30+ universities.

Rosemarie Truman, CEO of The Center for Advancing Innovation, led the efforts to assess the 4000+ inventions using a rigorous invention assessment model. She is now leading the challenge, in partnership with the Avon Foundation and the NCI, which she believes could change the dynamics of venture philanthropy and Federal intramural research commercialization for the long-term. “Outside of this challenge, a foundation such as the Avon Foundation will typically provide $150,000 to a Principal Investigator/inventor to further their research. Funding is committed for three years so, for 30 PIs, that equates to $13.5 million in total, or $4.5 million per year. But imagine a world in which just $250,000 – which is what has been provided by the Avon Foundation towards this challenge – can spur 30 start-ups to advance research in a particular therapeutic area. With $1 million, 120 start-ups could be launched through this program”, explains Rosemarie. “Imagine a 50% increase in intramural invention commercialization from Federal labs – such endeavours could really change the landscape of health.”

Marc Hurlbert, Executive Director of the Avon Foundation Breast Cancer Crusade (http://www.avonfoundation.org/causes/breast-cancer-crusade/) emphasized that the challenge is designed to accelerate and increase the volume of breast cancer inventions in development. Moreover, they hope to “spur economic growth and provide universities with a platform to develop their entrepreneurship-learning portfolios”.

**Figure:**
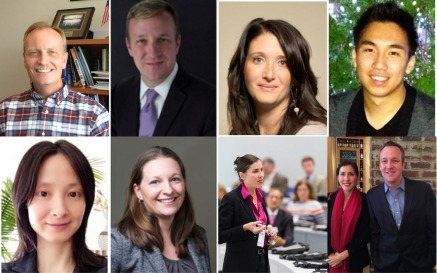
**Breast Cancer Start-up Challenge Team.** From left to right, top: Tom Stackhouse^1^, Marc Hurlbert^2^, Carolyn Ricci^2^, Jonathan Lui^3^; bottom: Youhong Wang^3^, Rose Freel^1^, Rosemarie Truman^3^, Rosemarie Truman^3^ and Marc Hurlbert^3. 1^Technology Transfer Center at the National Cancer Institute. ^2^Avon Foundation. ^3^The Center for Advancing Innovation.

A total of 200 teams received invitations to enter the contest, and 46 teams have been accepted with 431 people cumulatively participating within teams: http://www.breastcancerstartupchallenge.com/teams-participating.html. Challenge teams include but are not limited to students from the medical, business and/or law schools of Wake Forest University, University of North Carolina, Harvard, NYU, Rutgers, Ohio State, and the Icahn School of Medicine at Mount Sinai. The challenge has also attracted team members from outside the USA, including India, UK, Canada, New Zealand and The Netherlands. Each team must incorporate at least four disciplines: legal, medical/scientific, business and a seasoned entrepreneur. Illustrating the breadth of experience and expertise, the cumulative number of years of experience on all teams participating includes:

- 791 in start-ups
- 1523 in science/medicine
- 839 in consulting in a relevant field
- 1203 in life sciences business operations.

Tom Stackhouse, Associate Director of the Technology Transfer Center at the National Cancer Institute, said: “We are excited that so many high-quality teams have come together for this challenge. We are looking forward to seeing the outcome”.

DMM will provide readers with updates during each phase of the challenge, and will profile the winning team at the end of the contest, scheduled for June 27, 2014.

